# In Silico Destructive Activity of Phytochemicals From Ocimum sanctum Against the T9SS Proteins of Porphyromonas gingivalis

**DOI:** 10.7759/cureus.105127

**Published:** 2026-03-12

**Authors:** Vishnudas H, Anjali Chaudhary, Kennedy Babu, Manoj Margabandhu, Lakshminarayanan S, Jawahar Raman L

**Affiliations:** 1 Department of Periodontology, Mahatma Gandhi Postgraduate Institute of Dental Sciences, Puducherry, IND; 2 Department of Bioinformatics, Phytomatics Lab, Pondicherry University, Puducherry, IND

**Keywords:** anti-virulence, molecular docking, ocimum sanctum, periodontitis, pora, pore, porphyromonas gingivalis, rosmarinic acid, type ix secretion system (t9ss)

## Abstract

Periodontitis is a chronic inflammatory condition closely associated with the pathogenic activity of *Porphyromonas gingivalis *(*P. gingivalis*). Among its virulence components, the PorA and PorE proteins play essential roles in the type IX secretion system (T9SS), facilitating the release of gingipain-related factors that contribute to tissue destruction. Targeting these proteins may offer a promising therapeutic strategy. This study employed a molecular docking approach to evaluate phytocompounds from *Ocimum sanctum* (Tulsi) as potential antibacterial lead molecules against PorA and PorE. Ten Tulsi-derived compounds were chosen as ligands, and their binding affinities were compared with those of standard antibiotics, penicillin G, metronidazole, and doxycycline, using the Maestro module of the Schrödinger suite (v2023-2, Schrödinger, Inc., New York City, NY, USA). Absorption, distribution, metabolism, excretion, and toxicity (ADMET) evaluation was conducted using SwissADME (Molecular Modelling Group of the SIB Swiss Institute of Bioinformatics and the University of Lausanne, Lausanne, Switzerland), and protein-ligand interactions were visualized to determine molecular compatibility. Among the studied compounds, four (rosmarinic acid, eugenol, penicillin G, and metronidazole) showed inhibitory potential against PorE, while two (rosmarinic acid and penicillin G) demonstrated activity against PorA. Rosmarinic acid displayed the most favorable docking profile, with the highest binding energies for both PorA (ΔG = −3.501 kcal/mol) and PorE (ΔG = −5.728 kcal/mol), outperforming the reference antibiotics and the remaining phytochemicals. These results indicate that rosmarinic acid exhibits strong in silico potential to interfere with T9SS function by targeting PorA and PorE. The compound merits further investigation through in vitro and ex vivo studies as a promising natural inhibitor for managing pathogenic periodontal biofilms and disrupting T9SS-mediated virulence.

## Introduction

*Porphyromonas gingivalis* (*P. gingivalis*), a keystone pathogen in periodontitis, is highly pathogenic as a constituent of oral biofilm and has been associated with several systemic diseases [1]. The dysbiosis caused by the organism influences the host's immune response, which can affect the tissue destruction and extensive progression of the periodontal disease [2]. Therefore, several systemic and local treatment methodologies have been implicated in the elimination of *P. gingivalis* and other perio-pathogens [3]. *P. gingivalis*, along with periodontitis, has been implicated in various pathophysiologies, including diabetes, hypertension, preterm labor, and Alzheimer’s disease [4, 5]. Gingipains are cysteine proteases that make up more than 85% of the proteolytic activity of *P. gingivalis* [6].

Scaling and root planning, along with pocket elimination surgeries, are the most commonly used modes of treatment of periodontal disease [7]. Adjunct therapies like locally applied antibiotics and phytochemicals (organic compounds derived from plants) have been explored for maintaining and protecting periodontal health [8]. Plants having naturally synthesized compounds and antimicrobial properties may exert protective as well as host modulation properties [9]. Holy basil or *Ocimum sanctum *(Tulsi) is quite popular for its varied medicinal properties and ayurvedic remedies [10]. It has many useful phytochemicals such as eugenol, rosmarinic acid, flavonoids, and many terpenoids [11]. Previous studies have revealed that phytoconstituents present in tulsi are antimicrobial, antiprotozoal, anti-inflammatory, antipyretic, and neuroprotective [12]. Essential oils have been studied against various virulence factors of *P. gingivalis* and red complex bacteria. Various formulations like mouthwash, dentifrices, gels, and intracanal medicaments of tulsi are already being used for oral and periodontal diseases [13]. Comparable in vitro antimicrobial screening approaches have been used successfully in dental research to validate plant extracts before translational testing [14].

Precision and personalized medicines are the result of a paradigm shift in the treatment of various diseases. They use biological data to make decisions about prevention and treatment planning. Data can be taken at the molecular level, which concerns the genome, transcriptome, epigenome, proteome, and metabolites [15]. The type IX secretion system (T9SS) is a protein transport system found in Gram-negative bacteroidota, such as *Porphyromonas*, *Cytophaga*, *Capnocytophaga*, etc. In *Porphyromonas*, it secretes many virulence factors like gingipains and several enzymes that maintain its ecosystem and its pathological nature [16]. PorA and PorE are two newly discovered protein parts in T9SS systems that are essential for their normal functional characteristics [17, 18]. Some of the T9SS proteins are regulated by an extracytoplasmic function (ECF) sigma factor, PorX-PorY, SigP, and a cargo protein called PorA. PorE, which was initially called PG1058, has been suggested to have a role in linking periplasm and outer membrane components of T9SS. The mutation of PorE can lead to non-functional T9SS and certain defects in the characteristic pigmentation of the colony and proteolytic activity [18].

The PorA component, previously known as PGN_0123, is involved in the protein expression of genes concerning T9SS components; it can translocate to the cell surface without T9SS machinery. The N-terminal domain resembles the ligand-binding domain of FimH of *Escherichia coli*. The CTD has an NT sequence of 27 residues (1 to 27 signal peptides of a 246 AA chain) [17].

PorE can be divided into four domains: the tetratricopeptide repeat (TPR) domain, the beta-propeller domain, the carboxypeptidase regulatory domain (CRD)-like fold, and the OMP-C-like putative peptidoglycan-binding domain. The beta-propeller and TPR domains are the ones with protein-interactive functions that serve as a bridge between the periplasm and the T9SS OM components [18]. Despite the known and essential nature of T9SS proteins PorA and PorE, research on the use of phytotherapeutics against these components remains lacking. Therefore, for the first time, to the best of our knowledge, this research aimed to explore the potential of essential oils derived from *Ocimum sanctum* for their antimicrobial properties against the *P. gingivalis* proteins PorA and PorE as an alternative to existing chemical molecules used in periodontal therapy. The primary objective of this study was to evaluate the inhibitory potential of selected *Ocimum sanctum* phytocompounds against the T9SS proteins PorA and PorE of *P. gingivalis* using molecular docking approaches. Secondary objectives included (1) comparison of docking performance with standard antimicrobial agents (penicillin G, metronidazole, and doxycycline); (2) analysis of protein-ligand interaction profiles; and (3) in silico absorption, distribution, metabolism, excretion, and toxicity (ADMET) profiling of promising compounds. Compounds were considered to demonstrate favorable predicted binding activity if their Glide XP docking score was more negative than −3.0 kcal/mol and if they formed at least two specific interactions (hydrogen bonds or electrostatic contacts) with key residues of the binding pocket.

## Materials and methods

Protein retrieval and preparation 

**Figure 1:**
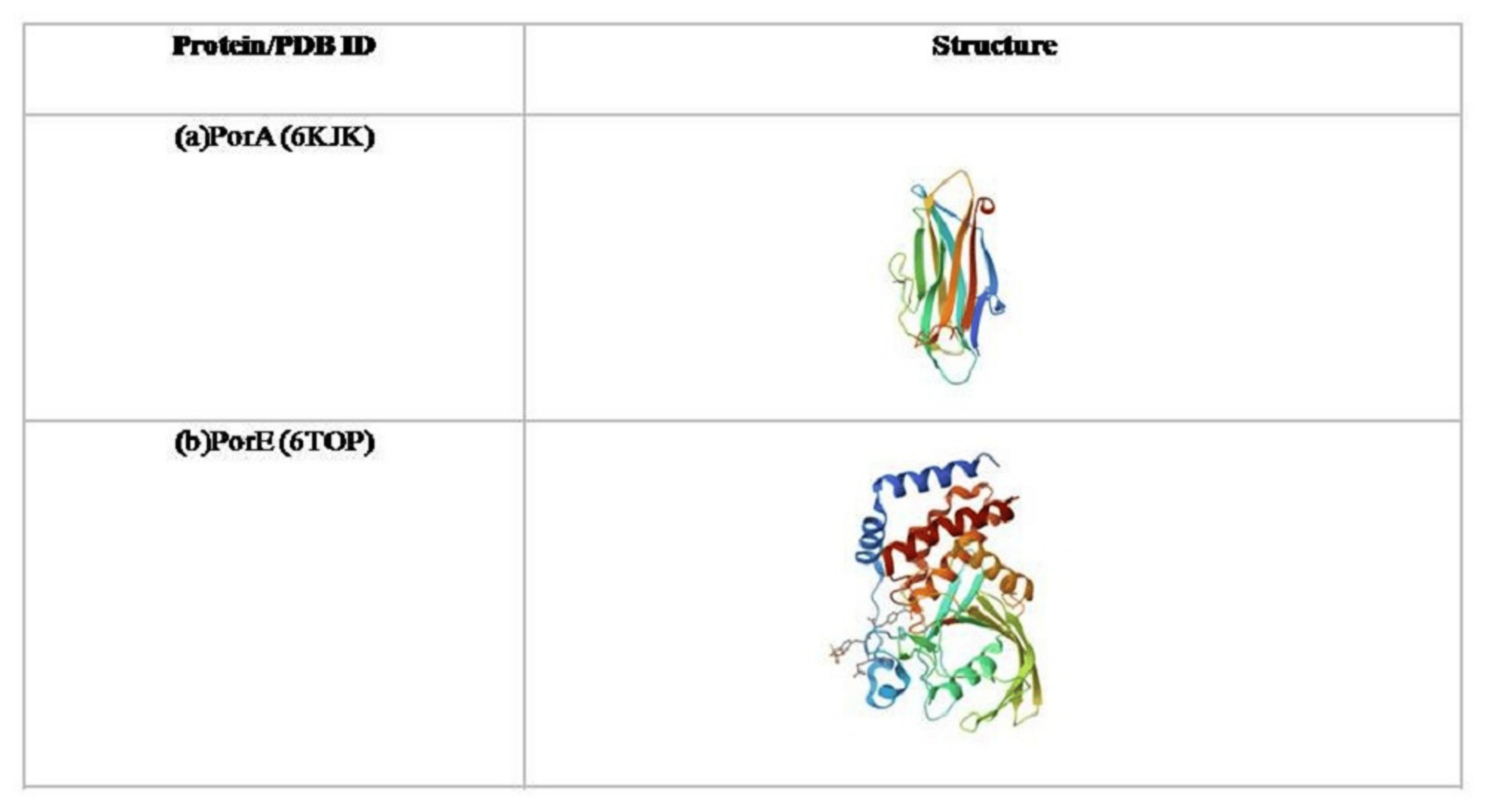
Three-dimensional structures of Porphyromonas gingivalis PorA and PorE proteins This figure illustrates the crystallographic structures of key type IX secretion system (T9SS) proteins from *Porphyromonas gingivalis*. Panel (a) shows the β-barrel–rich architecture of PorA (PDB ID: 6KJK), which is implicated in gingipain export and surface attachment. Panel (b) depicts the multi-domain conformation of PorE (PDB ID: 6TOP), a periplasmic component essential for T9SS stability and substrate translocation. The 3D structure of the target proteins (PorA and PorE) of *Porphyromonas gingivalis* was retrieved from the Protein Data Bank (PDB) with accession codes 6KJK and 6TOP. 6TOP had four domains, namely, TPR (25-149aa), WD40 (167-435aa), CRD (441-527aa) and OmpA_C_like (534-668 aa), of which OmpA_C_like domain, which had the putative peptidoglycan binding domain17 and bridges between the outer membrane and peptidoglycan, was selected for docking studies. Tables were created with Microsoft Word (Microsoft Corp., Redmond, WA, USA).

The 3D structures of the target proteins (PorA and PorE) of *P. gingivalis* were retrieved from the protein data bank (PDB) with accession codes 6KJK and 6TOP (Figure 1). As 6TOP had four domains, namely, TPR (25-149 aa), WD40 (167-435 aa), CRD (441-527 aa), and OmpA_C_like (534-668 aa), of which the OmpA_C_like domain, which had the putative peptidoglycan binding domain and bridges between the outer membrane and peptidoglycan, was selected for docking studies [18]. Prior to docking, the receptor protein structure was prepared using Maestro (Schrödinger Suite, v2023-2, Schrödinger, Inc., New York City, NY, USA), which involved deleting water molecules bound to the target protein, removing additional ligands, and adding hydrogen atoms. Further, the proteins were optimized by removing bound ligand molecules and heteroatoms, and energy minimization was performed using PyMOL (Schrödinger, Inc.) and Swiss-PdbViewer (Swiss Institute of Bioinformatics, Lausanne, Switzerland), respectively. Moreover, a grid was generated around the whole protein for 6KJK and the OMP-like C domain for 6TOP with dimensions of 10 Å × 10 Å × 10 Å centered on the geometric center of each binding region. Protocol validation was performed by re-docking a reference compound into the prepared receptor to confirm that the docking workflow reproduced known binding interactions.

Ligand preparation

**Figure 2:**
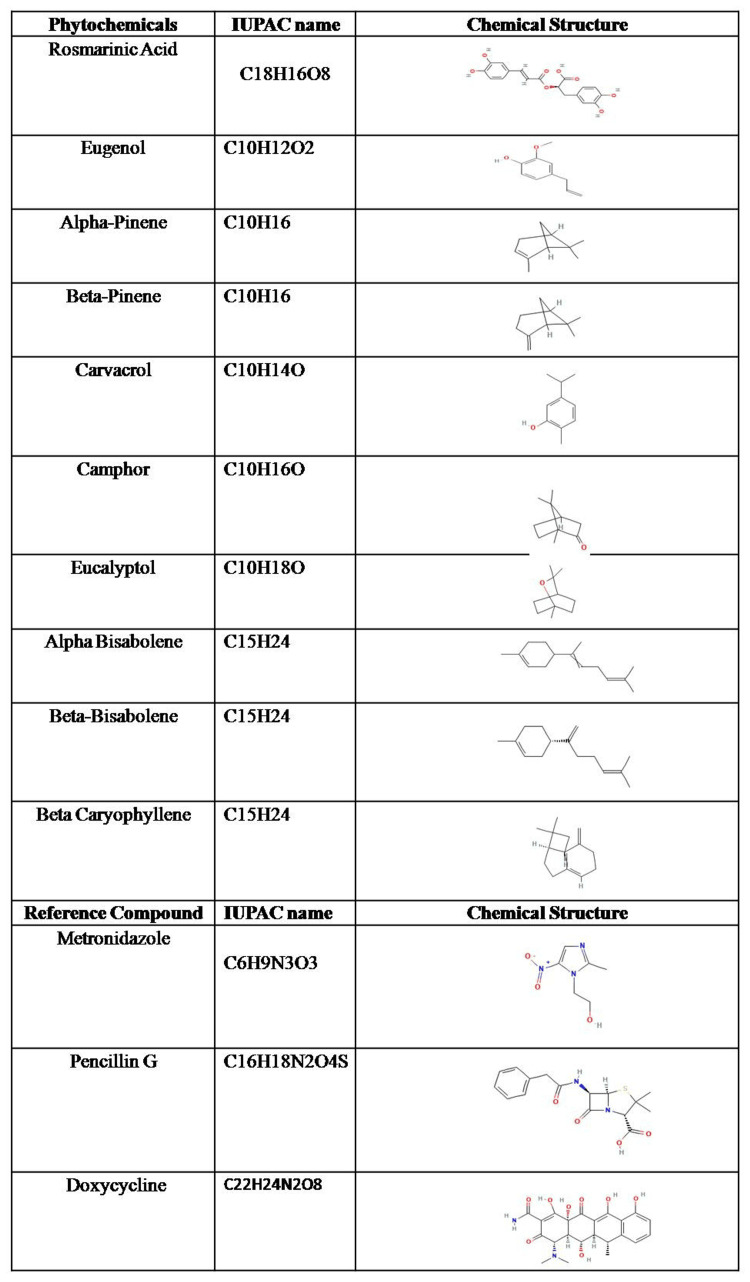
Phytochemicals and reference compounds used for molecular docking analysis This figure summarizes the chemical profiles of the 10 phytochemicals selected from *Ocimum sanctum*, along with the reference antimicrobial compounds used for comparison in the molecular docking study. For each compound, the table presents the common name, molecular formula (International Union of Pure and Applied Chemistry (IUPAC) molecular formula representation), and its corresponding two-dimensional chemical structure. The phytochemicals include rosmarinic acid, eugenol, alpha-pinene, beta-pinene, carvacrol, camphor, eucalyptol, alpha-bisabolene, beta-bisabolene, and beta-caryophyllene. Reference drugs included metronidazole, penicillin G, and doxycycline. These structures were retrieved and optimized prior to docking against PorA and PorE targets of *Porphyromonas gingivalis*. Three-dimensional chemical structures were retrieved from PubChem (National Center for Biotechnology Information, National Institutes of Health, Bethesda, MD, USA), which were saved in SDF/mol2 format and converted to PDBQT wherever needed. Tables were created and arranged with Microsoft Word (Microsoft Corp., Redmond, WA, USA).

Based on the literature review, 10 antimicrobial phytocompounds from *Ocimum sanctum*, aka *Ocimum tenuiflorum* [11, 19], were selected randomly without any pre-filtering and stratification, while doxycycline and metronidazole were taken as control drugs, as they are routinely used in the treatment of periodontal and oral infections [19-21]. Among phytochemical ligands, rosmarinic acid [22], eugenol [23], alpha-pinene [24], carvacrol [10], camphor [25], eucalyptol [26], and beta-caryophyllene [27] are shown to have known antimicrobial activities, and beta-bisabolene [28] was found to have anti-inflammatory activities. Beta-pinene is a potential perioceutic [29]. Further, the 3D chemical structures were retrieved from PubChem (National Center for Biotechnology Information, National Institutes of Health, Bethesda, MD, USA) (Figure 2), saved in SDF/MOL2 format, and converted to PDBQT format wherever required for docking analysis. Furthermore, ligand preparation was performed using the LigPrep module of the Schrödinger Suite (v2023-2; Schrödinger, Inc), with protonation states generated at physiological pH 7.0 ± 2.0 using Epik (Schrödinger, Inc.). All possible tautomers and stereoisomers were generated, resulting in 13 ligands expanding to 33 conformations in total.

Molecular docking and visualization

The prepared protein zip file containing grid information and prepared ligands (output file) was docked to analyze for its binding energy based on the receptor affinity of the ligand. The docking studies were done using the Glide package of Schrödinger Suite, where penalties and errors were eliminated by using extra precision (XP) docking. The top-scoring pose for each ligand was selected based on the Glide XP docking score (kcal/mol), which provides an estimate of binding affinity. Further, the protein-ligand interactions were screened to assess processed top-scoring outputs by comparing them with the docking scores of the reference molecules, and interacting amino acids were analyzed. Moreover, 2D and 3D interactions were visualized using Schrödinger and Protein Ligand Interaction Profiler (PLIP; ZBH Center for Bioinformatics, University of Hamburg, Hamburg, Germany), respectively.

ADMET analysis

The pharmacokinetic properties of the ligand molecules were predicted using the SwissADME online server (Molecular Modelling Group of the SIB Swiss Institute of Bioinformatics and the University of Lausanne, Switzerland) to quantitatively analyze the pharmacokinetic, physicochemical, water solubility, drug likeness, and lipophilicity properties. Further, the Lipinski’s rule was followed, which includes molecular weight ≤ 500 Da, ≤ 5 hydrogen bond donors, ≤ 10 hydrogen bond acceptors, and LogP ≤ 5. Predicted blood-brain barrier permeability and gastrointestinal absorption were also evaluated.

**Figure 3:**
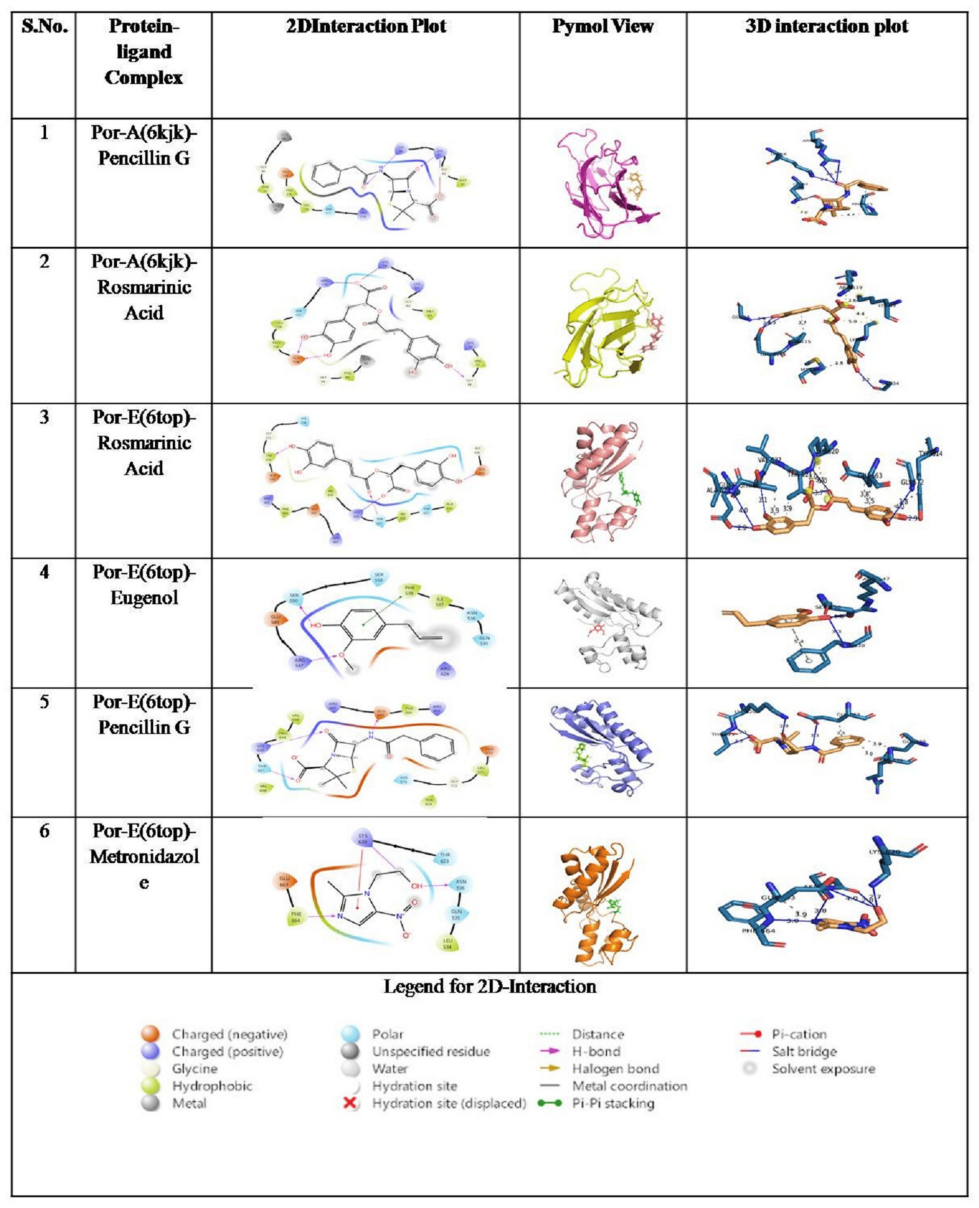
Molecular docking interaction profiles of phytochemicals and reference drugs with PorA (6KJK) and PorE (6TOP) This figure illustrates the interaction analyses of selected *Ocimum sanctum* phytochemicals and reference antimicrobial drugs with the PorA (PDB: 6KJK) and PorE (PDB: 6TOP) proteins of *Porphyromonas gingivalis*. For each protein–ligand complex, the 2D interaction plot highlights hydrogen bonding, hydrophobic contacts, polar interactions, metal coordination, salt bridges, and π–π stacking. PyMOL visualizations depict the overall orientation of each ligand within the protein binding pocket. The 3D interaction plots further show atomic-level docking conformations and key binding residues. Panel 1: PorA–penicillin G; Panel 2: PorA–rosmarinic acid; Panel 3: PorE–rosmarinic acid; Panel 4: PorE–eugenol; Panel 5: PorE–penicillin G; Panel 6: PorE–metronidazole. The legend below the figure summarizes the color codes and interaction symbols used in the 2D docking diagrams. 2D and 3D interactions were visualized using Schrödinger (Schrödinger, Inc., New York City, NY, USA) and Protein Ligand Interaction Profiler (PLIP; ZBH Center for Bioinformatics, University of Hamburg, Hamburg, Germany), respectively. Tables were created and arranged with Microsoft Word (Microsoft Corp., Redmond, WA, USA).

Furthermore, the interaction between protein-ligand complexes was determined by PLIP, which revealed 6KJK binding with distinct mechanisms. The interaction with penicillin G, one of the reference compounds (Figure 3), reflected a combination of hydrogen bonds and a salt bridge with surrounding hydrophobic stabilization. A salt bridge was formed between the carboxylate group of the beta-lactam ring and the positively charged side chain of Lys61, whereas the hydrogen bonds were formed between the H-bond acceptor carbonyl oxygen and the amide of Gly62. Additionally, the amide (N-H) of the side chain acts as an H-bond donor to the carbonyl group of Lys59. Besides the phytocompound, rosmarinic acid (Figure 3) displayed more hydrogen bonds with a wider binding pocket where the carboxylate group on the caffeoyl moiety forms a direct H-bond with the side chain of Ser117. Moreover, two hydroxy-phenyl (catechol) groups were also formed: one in the hydroxyl group at position 3 to the side chain of Glu114 and the terminal hydroxyl group on the cinnamoyl moiety with the amide side chain of Gly34.

For 6TOP, molecular docking showed the most favorable binding with rosmarinic acid, followed by penicillin, eugenol, and metronidazole. For rosmarinic acid (Figure 3), the C4 hydroxyl group in the dihydroxyphenyl-lactic acid moiety acts as an H-bond donor to the side chain of Glu635. The C4 hydroxyl group of the caffeic moiety acted as a donor to the side chain of Tyr614. The C3 group of the same ring acted as a donor for Gly663. Further, the residues such as Val618 and Val622, and charged residues like Arg661 and Glu663, were encapsulated in the hydrophobic skeleton. With Penicillin G (Figure 3), three hydrogen bonds were formed: one between the amide group and Glu663, a second between the beta-lactam carbonyl oxygen and the amide group of Thr621, and a last one between the carboxylate group and the side chain of Thr621, with an additional electrostatic interaction with Lys620. Perhaps the hydrophobic and aromatic stabilization was done by the phenylacetamido side chain, which was inserted in the hydrophobic subpocket lined by Phe664, Tyr614, and Leu571, while histidine contributed to nonpolar contact.

Eugenol (Figure 3) showed a hydroxy group acting as a donor to the side chain of Ser550, whereas the methoxy group formed a hydrogen bond with arginine. Further, the aromatic ring was stabilized by an alkyl interaction with Phe538, along with a charged residue, Arg629, presented in close proximity, indicating general electrostatic contact with Ile537, Asp536, and Glu535 surrounding the ligand. Metronidazole (Figure 3) displayed hydrogen bond and pi-pi stacking interactions with a charged residue, where the hydroxy side chain was the main polar anchor to which the hydroxyl group was the donor group of Asp536. Additionally, the nitro group was stabilized with an electrostatic or hydrogen bond interaction with positively charged Lys620, whereas an aromatic/π stacking interaction was seen with the nitroimidazole ring, which was perfectly oriented with the aromatic ring of Phe664. The binding pocket was shaped by residues including Thr623, Glu535, and Leu534.

## Results

In this study, we first tried to predict protein-ligand interactions by reducing the energy of ligands and estimating binding energies. Ligand binding to a particular protein receptor can cause enzyme inhibition by the ligand, which shows the possibility of biochemical interaction. (Table 1) The docking algorithm uses ligand activator and inhibitor properties with protein receptors and can link the drug's structure and cytotoxicity.

**Table 1:** Binding energy of protein-ligand complexes Rosmarinic acid had the highest binding energy compared to other ligands in both complexes.

Protein	Ligand	Binding Energy (kcal/mol)
PorE	Rosmarinic Acid	-5.728
	Pencillin G	-3.745
	Eugenol	-3.514
	Metronidazole	-3.322
PorA	Rosmarinic Acid	-3.502
	Penicillin G	-3.173

Based on the literature, 10 phytochemicals, namely, α-bisabolene, β-bisabolene, α-pinene, β-pinene, β-caryophyllene, camphor, carvacrol, eucalyptol, eugenol, and rosmarinic acid from *Ocimum sanctum*, were docked with target proteins 6KJK (PorA) and 6TOP (PorE). With standard drugs, metronidazole, doxycycline, and penicillin G were used as reference molecules. (Tables 2, 3). Of the 10 phytochemicals, eight compounds (α-bisabolene, β-bisabolene, α-pinene, β-pinene, camphor, carvacrol, eucalyptol, and β-caryophyllene) did not produce valid docking poses for either target under the applied XP precision settings. This may be attributable to their smaller molecular size, lower polarity, and limited capacity to form specific polar contacts within the binding pockets of PorA and PorE - both of which favour hydrogen bonding and electrostatic interactions.

**Table 2:** Key interactions of docked ligands with the PorE (6TOP) protein H-Bond (Donor): Ligand donates a hydrogen atom to the protein residue; H-Bond (Acceptor): Ligand accepts a hydrogen atom from the protein backbone or side chain; Electrostatic/ionic: Charge-based interaction between oppositely charged groups; Electrostatic anchor: Strong ionic contact stabilizing ligand orientation in the binding pocket; Hydrophobic interaction: Non-polar contacts involving aliphatic or aromatic residues; Hydrophobic encapsulation: Ligand aromatic/aliphatic regions surrounded by hydrophobic residues; π–π stacking: Aromatic ring–ring interaction between ligand and protein residues; π–Alkyl interaction: Interaction between ligand aromatic ring and protein alkyl chains; General polar/hydrophobic: Mixed polarity interactions involving both polar and non-polar residues; Backbone interaction: Contact involving the peptide backbone (N–H or C=O); Side-chain interaction: Contact involving amino-acid side-chain atoms.

Ligand	Functional Group/Moiety	Interaction Type	Key Interacting Residue(s)
Rosmarinic Acid	C-4 Hydroxyl (R-stereocenter)	H-Bond (Donor)	Glu635 (Side Chain)
	C-4 Hydroxyl (Caffeic acid)	H-Bond (Donor)	Tyr614 (Side Chain)
	C-3 Hydroxyl (Caffeic acid)	H-Bond (Donor)	Gly572 (Backbone)
	Carboxylate (COO−)	Ionic / Electrostatic	Lys620, Thr621
	Aromatic/Linker Skeleton	Hydrophobic Encapsulation	Val618, Val622, Arg661, Glu663
Penicillin	Carboxylate (COO−)	Electrostatic Anchor	Lys620
	Carboxylate (COO−)	H-Bond (Side Chain)	Thr621
	β-Lactam Carbonyl (C=O)	H-Bond (Acceptor from Backbone)	Thr621 (N−H)
	Side Chain Amide (N−H)	H-Bond (Donor to Backbone)	Glu663 (C=O)
	Phenylacetamido Ring/Chain	Hydrophobic/Aromatic	Phe664, Tyr614, Leu571, His574
Eugenol	Hydroxyl (HO-)	H-Bond (Donor)	Ser550
	Methoxy Oxygen (O−CH3​)	H-Bond	Arg547
	Aromatic Ring	π-Alkyl Stacking	Phe538
	Propenyl Chain/Aromatic Ring	General Hydrophobic	Ile537, Asn536, Gln535
Metronidazole	Nitroimidazole Ring	π−π Stacking	Phe664
	Hydroxylethyl OH	H-Bond (Donor)	Asn536 (Side Chain)
	Nitro NO2​ Oxygen	Electrostatic / H-Bond	Lys620
	Hydroxylethyl Chain/Ring	General Polar/Hydrophobic	Thr623, Gln535, Leu534

**Table 3:** Key Interactions of Docked Ligands With the PorA (6KJK) Protein Salt bridge: Ionic interaction between oppositely charged groups; H-Bond (donor): Ligand donates a hydrogen atom to the protein; H-Bond (acceptor): Ligand accepts a hydrogen atom from the protein; H-Bond (backbone): Hydrogen bond formed with the peptide backbone (N–H or C=O); H-Bond (side chain): Hydrogen bond formed with amino acid side-chain atoms; Hydrophobic contacts: Non-polar interactions with aliphatic or aromatic residues; Van der Waals: Weak, nonspecific attractive interactions within close proximity; Electrostatic contribution: Charge-based stabilizing interaction due to nearby charged residues; Aromatic contacts / π-interactions: Non-covalent interactions involving aromatic rings.

Ligand	Functional Group/Moiety	Interaction Type	Key Interacting Residue(s)
Penicillin	Carboxylate (COO−)	Salt Bridge (Ionic Anchor)	Lys61 (Side Chain)
	β-Lactam Carbonyl (C=O)	H-Bond (Acceptor from Backbone)	Gly62 (N−H)
	Side Chain Amide (N−H)	H-Bond (Donor to Backbone)	Lys59 (C=O)
	Phenyl Ring / Alkyl Spacer	Hydrophobic Contacts	Pro115, Leu116
	Core Structure	General Van der Waals	Cys96, Gly94, Phe93, Met63
Rosmarinic Acid	Carboxylate (COO−, Caffeoyl Moiety)	H-Bond (Side Chain)	Ser117
	OH at position 3 (R-stereocenter)	H-Bond (Donor)	Glu114 (Backbone/Side Chain)
	Terminal OH (Cinnamoyl Moiety)	H-Bond (Donor to Backbone)	Gly34 (N−H)
	Aromatic / Aliphatic Portions	Hydrophobic Contacts	Pro115, Leu116, Cys92
	Overall Structure	Electrostatic Contribution	Arg119, Lys59 (Proximity)

For 6KJK, the molecular docking analysis revealed rosmarinic acid to have a binding energy of -3.502 kcal/mol when compared with penicillin G, which displayed -3.173 kcal/mol, while other compounds didn’t dock. Perhaps, for 6TOP, rosmarinic acid was found to have a binding energy of -5.728 kcal/mol when compared to penicillin G, eugenol, and metronidazole, which had binding energies of -3.745 kcal/mol, -3.514 kcal/mol, and -3.322 kcal/mol, respectively. For 6KJK, the binding activity was seen in the following order: rosmarinic acid > penicillin G, whereas for 6TOP, it was rosmarinic acid > penicillin G > eugenol > metronidazole.

ADMET analysis identified rosmarinic acid to have excellent oral availability but poor GI absorption and no crossing of the blood-brain barrier. Penicillin G also showed poor GI absorption and didn't cross the blood-brain barrier. Both eugenol and metronidazole had good GI bioavailability, but only metronidazole showed crossing of the blood-brain barrier. These differences should be interpreted in the context of local periodontal drug delivery, where systemic absorption is not the primary route of administration.

Furthermore, the interaction between protein-ligand complexes was determined by PLIP, which revealed 6KJK binding with distinct mechanisms. The interaction with penicillin G, one of the reference compounds (Table 3), reflected a combination of hydrogen bonds and a salt bridge with surrounding hydrophobic stabilization. A salt bridge was formed between the carboxylate group of the beta-lactam ring and the positively charged side chain of Lys61, whereas the hydrogen bonds were formed between the H-bond acceptor carbonyl oxygen and the amide of Gly62. Additionally, the amide (N-H) of the side chain acts as an H-bond donor to the carbonyl group of Lys59. Besides the phytocompound, rosmarinic acid (Table 3) displayed more hydrogen bonds with a wider binding pocket where the carboxylate group on the caffeoyl moiety forms a direct H-bond with the side chain of Ser117. Moreover, two hydroxy-phenyl (catechol) groups were also formed: one hydroxyl group at position 3 to the side chain of Glu114 and a terminal hydroxyl group on the cinnamoyl moiety with the amide side chain of Gly34.

For 6TOP, molecular docking showed the most favorable binding with rosmarinic acid, followed by penicillin, eugenol, and metronidazole. For rosmarinic acid (Table 3), the C4 hydroxyl group in the dihydroxyphenyl-lactic acid moiety acts as an H-bond donor to the side chain of Glu635. The C4 hydroxyl group of the caffeic moiety acted as a donor to the side chain of Tyr614. The C3 group of the same ring acted as a donor for Gly663. Further, the residues such as Val618 and Val622, and charged residues like Arg661 and Glu663 were encapsulated in the hydrophobic skeleton. With penicillin G (Table 3), three hydrogen bonds were formed: one between the amide group and Glu663, a second between the beta-lactam carbonyl oxygen and the amide group of Thr621, and a last one between the carboxylate group and the side chain of Thr621, with an additional electrostatic interaction with Lys620. Perhaps the hydrophobic and aromatic stabilization was done by the phenylacetamido side chain, which was inserted in the hydrophobic subpocket lined by Phe664, Tyr614, and Leu571, while histidine contributed to nonpolar contact.

Eugenol (Table 3) showed a hydroxy group acting as a donor to the side chain of Ser550, whereas a methoxy group formed a hydrogen bond with arginine. Further, the aromatic ring was stabilized by an alkyl interaction with Phe538, along with a charged residue, Arg629, presented in close proximity, indicating general electrostatic contact with Ile537, Asp536, and Glu535 surrounding the ligand.

Metronidazole (Table 3) displayed hydrogen bond and pi-pi stacking interactions with a charged residue, where the hydroxy side chain was the main polar anchor to which the hydroxyl group was the donor group of Asp536. Additionally, the nitro group was stabilized with an electrostatic or hydrogen bond interaction with positively charged Lys620, whereas an aromatic/pi stacking interaction was seen with the nitroimidazole ring, which was perfectly oriented with the aromatic ring of Phe664. The binding pocket was shaped by residues including Thr623, Glu535, and Leu534.

## Discussion

Over the past few years, phytotherapeutics have been of growing interest in the prevention and treatment of periodontal disorders. Medicinal plants, which are used in Indian and Chinese traditional medicine, are being researched for targeted therapy for periodontopathogens such as *P. gingivalis*, *Tannerella forsythia*, and *Treponema denticola*. *Ocimum sanctum* has a variety of essential oils and antimicrobial compounds, which are used [30]. Rosmarinic acid, one of the phytoconstituents present in *Ocimum sanctum*, has been the focus of many antimicrobial and anti-oncogenic properties. It is a constituent part of many culinary herbs, namely rosemary, thyme, oregano, lemon balm, perilla of the mint family, and the sage plant. It regulates oxidative stress, apoptosis, and the cell cycle [31]. *Rosmarinus officinalis* extract, which contains rosmarinic acid, has been shown to have in situ antimicrobial properties against initial oral biofilms. There were significantly lower microbial counts in the treated patients through wearable splints containing rosmarinic extract. The authors have highlighted the need for using the extract against *P. gingivalis* and *Prevotella intermedia*. Rosmarinic acid has been found to have antifungal biofilm properties by inhibiting mitochondrial activity and causing a slight reduction in proteases with a decrease in membrane integrity in *Candida *species. Rosmarinic acid has also shown antibiofilm properties against *Streptococcus mutans*.

In silico research provides crucial and effective steps to combat and rule out unproductive compounds. This also saves us cost and time without doing in vitro tests, which makes it a diligent choice for preliminary research. The results of the present study aligned with the study that showed the antimicrobial properties of an herbal tincture containing rosmarinic acid [31]. Docking of the compound may play an important role in the regulation of T9SS proteins. With PorE (6TOP), rosmarinic acid had a greater span in occupying the pocket by multiple hydrogen bonds with Tyr614, Glu635, and Gly572, which showed hydrophilic specificity of the protein. Metronidazole can be seen anchored with π-interaction with eugenol, which was seen to have bound with π alkyl contacts, a hydrophobic interaction, neither of which was as strong as rosmarinic acid. With PorA (6KJK), rosmarinic acid had been shown to have a lower affinity binding than PorE. So, keeping in mind the above results, PorE can be used as a druggable target. The above results revealed the importance of phytochemicals and their derivatives, which could be indispensable in the treatment of periodontitis and other oral diseases. Although in our study, many compounds from *Ocimum sanctum *did not dock with the selected proteins, rosmarinic acid holds significant activity against the PorE protein when compared to penicillin G.

Limitations of the study

This study has several important limitations that must be acknowledged. First, the ligand selection was based on a literature review without the application of virtual screening prefilters or objective physicochemical criteria; this approach, while broad, may not represent the optimal set of phytochemical candidates. Second, the docking protocol was not formally validated through re-docking of a co-crystallized ligand or cross-docking benchmarking, which limits confidence in the reproducibility of pose predictions. Third, the reported docking scores are modest in magnitude, and their biological relevance as inhibitory thresholds has not been experimentally verified. Fourth, no molecular dynamics simulations were performed to assess binding stability over time, and no rescoring with orthogonal methods was applied. Fifth, the ADMET analysis was performed using a web-based predictive tool; the results reflect systemic pharmacokinetic predictions, which may not fully apply to the local periodontal delivery context where most of these compounds would be administered. Sixth, the in silico predictions of PorA and PorE inhibition have not been corroborated by in vitro functional assays such as minimum inhibitory concentration (MIC) determination, gingipain activity assays, or biofilm disruption assays. Future studies should address these limitations through experimental validation using in vitro and ex vivo models, docking protocol validation, and molecular dynamics simulations to strengthen the translational relevance of these findings.

## Conclusions

Precision and personalized medicine have resulted in a paradigm shift in the treatment of various diseases. Over the past few years, phytotherapeutics from medicinal plants used in traditional Indian and Chinese medicine have been researched for targeted therapy for periodontopathogens such as P. gingivalis. PorA and PorE are two newly discovered protein parts in the T9SS system, which are essential for its normal functional characteristics in virulence factor expression. In the present study, the results aligned with the in vitro studies, which showed antimicrobial properties of rosmarinic acid, and it could be a potential lead molecule against T9SS targets. Also, the PorE had a druggable target in which rosmarinic acid showed appreciable molecular docking and binding energy when compared to standard antimicrobials used in periodontics.

Prioritized next steps should include (1) in vitro validation through MIC determination, gingipain activity assays, and biofilm disruption assays; (2) orthogonal in silico validation through molecular dynamics simulation and binding free energy rescoring; and (3) assessment of rosmarinic acid within local periodontal delivery formulations. These studies will be essential before any translational claims can be made regarding the therapeutic potential of rosmarinic acid in periodontitis management.
